# IFN-I exacerbates the inflammatory response of epithelial cells to *Chlamydia trachomatis* infection by enhancing TLR3 expression

**DOI:** 10.1128/mbio.00527-26

**Published:** 2026-06-15

**Authors:** Chongfa Tang, Xiaonan Cai, Béatrice Niragire, Félix V. Louchez, Yaël Victoria Levy-Zauberman, Agathe Subtil, Yongzheng Wu

**Affiliations:** 1 Institut Pasteur, Université Paris Cité, UMR3691 CNRS, Cellular biology of microbial infection 27058, Paris, France; 2 Sorbonne Université, Collège doctoral, Paris, France; 3 National Vaccine and Serum Institute 111611, Beijing, China; 4 Lanzhou Institute of Biological Products 38042, Lanzhou, Gansu Province, China; 5 Service de Chirurgie Gynécologique, Institut Mutualiste Montsouris, Paris, France; Duke University School of Medicine, Durham, North Carolina, USA

**Keywords:** *C. trachomatis*, type I interferon, inflammation, interleukin 6, synergy, toll-like receptor 3, epithelial cells, female genital tract

## Abstract

The inflammation induced by *Chlamydia trachomatis* infection in the female genital tract can have severe consequences. Recent observations in women and mice infected with *Chlamydia* suggest that type I interferon (IFN-I) may have deleterious effects. This study aimed to elucidate the consequences of IFN-I production on *C. trachomatis*-induced inflammation in epithelial cells and the molecular pathway(s) involved. We showed that the combination of IFN-I and *Chlamydia* resulted in a stronger induction of inflammation than *Chlamydia* alone, while IFN-I alone had no effect. Inhibiting AKT and mTOR, but not silencing *STAT1*, significantly attenuated the synergistic effect between IFN-I and bacteria on inflammation. Inhibition of ERK also blocked this synergistic effect, although ERK was not activated by IFN-I. We hypothesized that IFN-I enhanced the expression of a pathogen recognition receptor of the host, thereby potentiating detection of the bacteria and the subsequent inflammatory response. IFN-I, but not *C. trachomatis* infection, increased the expression of Toll-like receptor 3 (TLR3), and silencing or knocking-out *TLR3* prevented the synergistic effect between infection and IFN-I. Furthermore, our data support the presence of dsRNA in infected cells and the activation of the MAPK/ERK and AP-1/ATF2 signaling cascades downstream of TLR3. Taken together, our data demonstrate that IFN-I exacerbates the host inflammatory response triggered by *Chlamydia* by increasing TLR3 expression and activation, leading to hyperinflammation. The identified signaling cascades represent potential targets for therapeutic intervention to limit tissue damage upon *Chlamydia* infection.

**IMPORTANCE:**

The effect of the production of IFN-I upon infection by *Chlamydia trachomatis* is not well understood. We showed that IFN-I exacerbated *Chlamydia*-induced inflammation in epithelial cells. This synergy was mediated by the IFN-induced upregulation of Toll-like receptor 3 (TLR3) expression, which facilitated sensing of *Chlamydia* and amplified the inflammatory response. We identified the signaling cascades involved upstream and downstream of TLR3 signaling. By exacerbating the pro-inflammatory response of epithelial cells, IFN-I might contribute to the hyperinflammation experienced by some individuals. The signaling pathways we uncovered can serve as a starting point for novel therapeutic strategies to alleviate tissue damage upon *Chlamydia* infection.

## INTRODUCTION

Infection by the obligate intracellular bacterium *Chlamydia trachomatis* is the leading cause of sexually transmitted infections of bacterial origin (1). The bacteria multiply principally in epithelial cells of the reproductive tract of men and women. In women, the bacteria can ascend from the vagina to the cervix, the endometrium, and the fallopian tubes. Epithelial cells from these tissues constitute the first line of defense against *C. trachomatis* infection. In particular, they sense *Chlamydia* through pattern recognition receptors (PRRs) (2–5), distributed either on the cell surface (e.g., Toll-like receptor 2 [TLR2], 4, and 6), in the cytoplasm (e.g., NODs), or in endosomes (e.g., TLR3, 7, 9) (6). Activation of these PRRs triggers signaling cascades that eventually lead to the expression of genes involved in innate immunity, such as pro-inflammatory cytokines and chemokines. Among those, interleukin 6 (IL6) is one of the major pro-inflammatory cytokines associated with *Chlamydia* infection (7–9). Inflammation contributes to the resolution of infection. However, in some women, it can result in tissue damage and scar formation, eventually causing severe consequences, such as pelvic inflammatory disease and tubal infertility.

Type I interferon (IFN-I) belongs to a group of cytokines that activate several signaling cascades, including Janus kinase 1 (JAK1)/tyrosine kinase 2 (TYK2) and downstream signal transducer and activator of transcription 1 (STAT1), mitogen-activated protein kinase (MAPK) kinase/ERK, phosphoinositide 3-kinase (PI3K)/AKT/p38, and PI3K/AKT/mammalian target of rapamycin (mTOR). These signaling cascades converge toward the induction of expression of interferon-stimulated genes (10). The best-known members of this family, IFNα and β, exert important roles in infectious diseases. While their protective properties against viral infections are well documented, the consequences of their production during bacterial infections are less studied (11, 12). IFN-I production appears to be beneficial to the host during infections by *Streptococcus pyogenes* (13), *Legionella pneumophila* (14, 15), *Bacillus anthracis* (16), *Shigella flexneri*, and *Salmonella typhimurium* (17, 18).

IFN-I is synthesized by many cell types, including immune cells. It is secreted by epithelial cells of the female genital tract (FGT) upon *C. trachomatis* infection both in humans and mice, including in an organoid model of infection (19–23). Early works have reported an inhibitory effect of IFN-I on *C. trachomatis* and *C. pneumoniae* replication in epithelial cells (24, 25). However, mice with IFN receptor gene knocked out (IFNAR-KO mice) or deficient for IFN-I signaling cleared *C. muridarum* infection earlier than control mice and developed less oviduct pathology (26). In addition, anti-IFN-I neutralizing antibodies also inhibited bacterial replication and subsequent tissue damage in the FGT of *C. muridarum*-infected mice (22). These data indicate that IFN-I production favors bacterial proliferation and tissue damage in a mouse model of *Chlamydia* infection. Furthermore, retrospective studies showed that both ascending *C. trachomatis* infection in the FGT of patients and *Chlamydia* infection in patients with pelvic inflammatory disease correlated with increased levels of interferon-inducible protein 10 (IP10), a cytokine produced in response to IFN-I exposure (27, 28). We also observed increased IP10 production by primary epithelial cells exposed to *C. trachomatis* infection (9).

The potential influence of IFN-I on the outcome of *C. trachomatis* infection prompted us to characterize its effect on epithelial cells, which constitute the first sentinels alerting to infection in the genital tract. The production of IL6 was markedly enhanced by exposure to IFN-I. By characterizing the molecular mechanism underlying the synergistic effect between IFN-I and *C. trachomatis* infection, we uncovered the central part played by the PRR TLR3.

## MATERIALS AND METHODS

### Cells

The human cervical epithelial cell line HeLa was from ATCC (Virginia, USA). The cells were grown in Dulbecco’s Modified Eagle’s Medium (DMEM) supplemented with GlutaMAX (Gibco, France) and 10% heat-inactivated fetal calf serum (FCS).

Primary epithelial cells were isolated from ecto-cervical explants of patients undergoing hysterectomy as described previously (9). The cells were cultured in keratinocyte serum-free medium supplemented with 5 µg/L of human recombinant epidermal growth factor and 50 mg/L bovine pituitary extract (K-SFM, Thermo Fisher Scientific, #10144892).

### Preparation of TLR3-KO cell line

TLR3-KO HeLa cells were established as described (29). In brief, 0.5 μg of pSpCas9(BB)−2A-Puro (PX459) V2.0 plasmid (Addgene #62988) containing guide RNA (gRNA) duplex sequence of human TLR3 (F: 5′-CACCGTACCAGCCG CCAACTTCACA-3′; R: 5′-AAACTGTGAAGTTGGCGGCTGGTAC-3′) inserted with BbsI enzyme site was transfected into pre-adhered HeLa cells (0.15 × 10^6^ cells/well, 24-well plate) using 1 μL of jetPRIME (Polyplus). Four hours later, fresh medium was replaced containing puromycin (2 μg/mL), and cells were incubated for 24 h. The surviving cells were detached, counted, and serially diluted to collect individual clones in 96-well plates.

To detect the indel mutation of TLR3, genomic DNA was extracted after expanding the individual clones, using DNeasy Blood and Tissue Kit (Qiagen). The gRNA-containing region (~700 bp) of the TLR3 gene was amplified (95°C for 30 s, 55°C for 30 s, 72°C for 30 s, ×35 cycles) from genomic DNA using GoTaq qPCR system (Promega) and T100 Thermal Cycler (Bio-Rad). The primers for PCR are listed in Table S1, and the primer for sequencing was 5′-GACTTTTGTCACGACTTCAC-3′. Analysis of the sequence using Tracking of Indels by Decomposition (TIDE, https://tide.nki.nl/) revealed the presence of 5 bp (two alleles) and 6 bp (one allele) deletions, consistent with the presence of three copies of the TLR3-containing chromosome 4 in HeLa cells (30).

### Bacterial preparation and infection

*C. trachomatis* LGV-L2 (434/Bu/ATCC), LGV-L2^IncD^mCherry, LGV-L2^IncD^GFP (stably expressing the fluorescent protein mCherry and GFP, respectively) (31), and the plasmid-less 25667R strain (32) were propagated in HeLa cells and purified as described (33). Purified elementary bodies were stored in 220 mM sucrose, 10 mM sodium phosphate (8 mM Na_2_HPO_4^−^_ 2 mM NaH_2_PO_4_), and 0.50 mM L-glutamic acid (SPG buffer) at −80°C. For infection, adhered cells were incubated with bacteria suspended in DMEM containing 10% FCS for different times (depending on the experiments) before harvesting the samples. Unless otherwise specified, the infections in HeLa cells were conducted with a multiplicity of infection (MOI) of 2, and five times more bacteria were used in experiments with primary cells (9) to reach >85% of cells being infected.

### IFN treatment

IFN-I (Biogen Idec) at 2.5 ng/mL final concentration (34, 35) was added to the culture medium at the same time as the bacteria, unless specified otherwise.

### Flow cytometry

Cells (0.3 × 10^6^ cells/mL) were seeded in 12-well plates. Twenty-four hours post IFN-I (Biogen Idec) treatment (2.5 ng/mL) and/or *Chlamydia* infection, the cells were incubated with 5 μg/mL brefeldin A solution (Biolegend #420601) for 6 h to block cytokine secretion. In certain experiments, the cells were pre-treated with pharmacological inhibitors for 1 h before bacterial infection and/or IFN-I treatment. Cells were then detached with 0.5 mM EDTA in PBS, fixed by 2% PFA/2% sucrose (wt/vol) in PBS for 20 min at room temperature, followed by quenching with 50 mM NH_4_Cl in PBS for 10 min. The cells were then permeabilized with 0.3% (vol/vol) Triton X-100 in PBS for 10 min and blocked in 1% bovine serum albumin (BSA) in PBS for 1 h, followed by incubation in PBS, and 0.1% BSA with or without 1/40 dilution of PE/Cyanine7-conjugated anti-human IL6 (Biolegend, #501119) for 1 h. After washing, the cells were resuspended in PBS and analyzed by CytoFLEX flow cytometer (Beckman Coulter). Unstained uninfected cells were used as a gating control. The data were analyzed using FlowJo (version 10.0.7).

### siRNA treatment

One and a half microliter siRNA (10 μM stock) was mixed with 0.75 μL Lipofectamine iMax (Invitrogen) in 50 μL of DMEM media (Gibco) for 10 min at room temperature. The mixture was added to the culture dish before adding cells (0.15 × 10^6^ cells/well for 24-well plate) suspended in 0.45 mL DMEM/FCS medium, mixed and incubated for 24 h before treatment or infection.

### Immunofluorescence

HeLa cells or p65-GFP expressing HeLa cells (36) were seeded on coverslips in a 24-well culture dish (0.15 × 10^6^ cells/well). The cells were treated with recombinant human IL1β (10 ng/mL, Thermo Fisher Scientific), IFNβ (2.5 ng/mL), and/or infected with *Chlamydia* strains (MOI = 1) as described in the legends of the figures. The cells were then fixed with 4% PFA and 4% sucrose for 20 min at room temperature, followed by quenching with 50 mM NH_4_Cl in PBS for 10 min. The cells were then permeabilized with 0.3% (vol/vol) Triton X-100 and 0.1% (wt/vol) BSA. Inclusions were stained using rabbit antibodies against the inclusion protein Cap1 (generated by the laboratory [37]) and secondary anti-rabbit Alexa Fluor 647 antibody. DNA was stained using 0.5 µg/mL Hoechst 33342 (Thermo Fisher Scientific) in PBS containing 0.1% BSA. dsRNA was detected using primary monoclonal J2 antibody (Scicons, #RNT-SCI-10010200) at a dilution of 1/50 and secondary anti-mouse Alexa Fluor 488 antibody. A duplicate infected coverslip was treated with 100 U/mL RNase-III (Ambion, AM2290) in PBS for 30 min with gentle agitation (20 rpm/min) at room temperature after fixation (38), followed by immunostaining. Coverslips were mounted on slides in a Mowiol solution. Images were acquired on an Axio Observer Z1 microscope equipped with an ApoTome module (Zeiss, Germany) and a 63× Apochromat lens. Images were taken with an ORCA-flash4.0 LT camera (Hamamatsu, Japan) using the software Zen.

### Quantification of dsRNA fluorescent signals

All segmentations were performed using Cellpose (39) pretrained model “cyto” with “diameter” parameter set to “0.” Cells were segmented based on the green/dsRNA staining and UV/DAPI channels. Inclusions were segmented based on the red/mCherry channel. Cell nuclei and inclusions appeared in the UV/DAPI channel (DNA stain). Segmentation of this channel using Cellpose resulted in masks corresponding both to inclusions and cell nuclei. The different steps described below were automatically performed using a Fiji macro script (40). Regions of interest (ROI) corresponding to inclusions and cells were respectively defined using the inclusion and cell masks from Cellpose. For each cell ROI, the macro checked each inclusion ROI one by one to see if there was an overlap with the cell ROI. This was done by selecting the intersection of both ROI (“AND” function of the ROI Manager) and by checking if it was not empty (selection type > −1). Cells were considered infected if they overlapped with at least one inclusion. Then areas of each cell corresponding to nuclei or inclusions were set to intensity values of 0 by subtracting the DAPI and inclusions masks from Cellpose. The total intensity in the cytoplasm in the dsRNA channel was then measured for each cell, and the values were divided by the mean of the uninfected cells to normalize across conditions. Finally, for each field, two composite pictures of DAPI, mCherry/Cap1, and dsRNA channels were saved, displaying either infected or non-infected cells segmentation for postprocessing verification (see Fig. S6A).

### RT-PCR, PCR, and quantitative PCR

Adhered cells (0.15 × 10^6^ cells/ml) in 24-well plates were treated with the indicated concentration of recombinant IFN-I, and/or infected with *C. trachomatis*, or with polyinosine-polycytidylic acid (polyI:C, InvivoGen, #tlrl-picw and #tlrl-pic), as synthetic agonists of TLR3 (41). Total RNAs were isolated 24 h post-IFN-I treatment, 40 h post-infection (hpi), or 16 h after polyI:C treatment with the RNeasy Mini Kit (Qiagen), and RNA concentrations were determined with a spectrophotometer NanoDrop (Thermo Fisher Scientific). Reverse transcription (RT) was performed using the M-MLV Reverse Transcriptase (Promega), and quantitative PCR (qPCR) was undertaken on the complementary DNA with LightCycler 480 system using SYBR Green Master I (Roche). Data were analyzed using the 2^−ΔΔCt^ method with the *actin* gene as a control gene, and results were presented as relative quantity compared to untreated or uninfected control cells.

The primers for PCR are shown in Table S1.

### Western blot

Adhered cells (0.2 × 10^6^ cells/mL) in 12-well plates were treated with the indicated concentration of recombinant IFN-I and/or infected with *C. trachomatis* for 24 h, with IFN-I in the absence or presence of SB203580 for 24 h, or with recombinant human TNFα for 30 min. Cells were then lysed in urea buffer (30 mM Tris, 150 mM NaCl, 8 M urea, 1% SDS, pH = 8.0) containing protease and phosphatase inhibitors (Roche, #11873580001 and #04906845001). Equal volumes of cell lysates were subjected to SDS-PAGE, transferred to polyvinylidene difluoride membranes, and immunoblotted with primary antibodies diluted in PBS containing 5% (wt/vol) fat-free milk and 0.01% (vol/vol) Tween-20. Primary antibodies used were rabbit anti-human STAT1 (#9172), ERK (#4695), p38 (#9212), AKT (#9272), rabbit anti-human phosphorylated STAT1 (#9167), ERK (#4370), p38 (#9215), AKT (#9275), and mTOR (#2971), which were purchased from Cell Signaling, rabbit antibodies against the heat shock protein 60 of *Chlamydia* (generated by the laboratory [42])*,* and mouse anti-human β-actin (Sigma, #A5441). Immunoblots were analyzed using horseradish peroxidase secondary antibodies (Abliance), and chemiluminescence was analyzed on a ChemiDoc Touch Imaging System (Bio-Rad).

### Statistical analysis

The experimental data were analyzed using Prism10 (GraphPad). Paired Student’s *t*-tests were used for two-group comparisons when the sample size was less than 8. When the sample size was 8 or more, one-way analysis of variance (ANOVA) with Tukey procedure was applied for multiple comparisons, as indicated in the legends.

## RESULTS

### IFN-I enhances the inflammatory response to *C. trachomatis* infection

**Fig 1:**
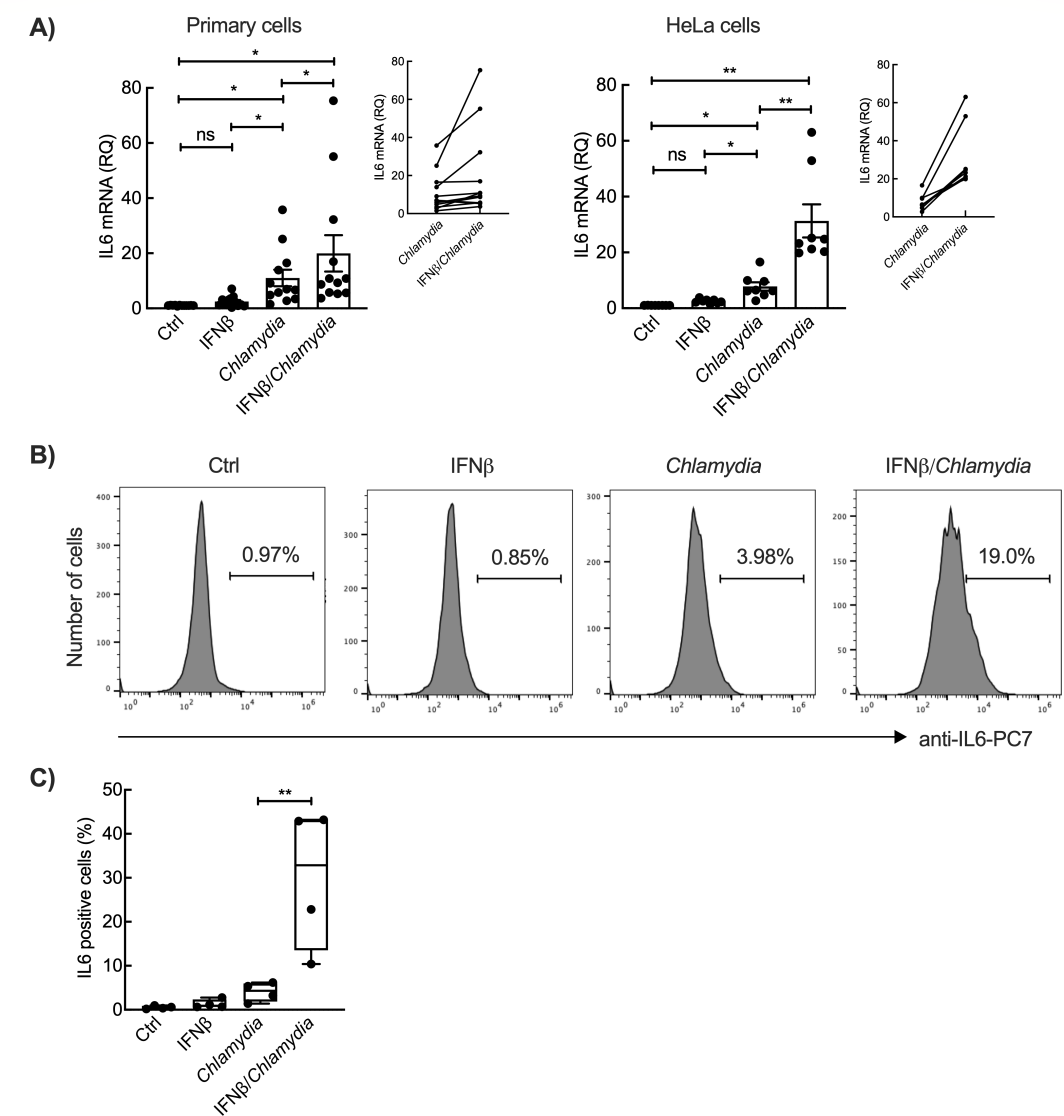
IFNβ enhances the inflammatory response of epithelial cells to *C. trachomatis* infection. (**A**) Primary cervical epithelial cells (left panel) or HeLa cells (right panel) were incubated with IFNβ for 24 h, or with *C. trachomatis* for 40 h in the absence or presence of IFNβ. The expression of inflammatory cytokines IL6 was detected by quantitative PCR. IL6 transcript was measured by real‐time RT-qPCR and normalized to actin transcript following the 2^−ΔΔCt^ method. The data are presented as relative mRNA levels compared to uninfected cells; five (for primary cells) or eight (for HeLa cells) independent experiments are shown, each measurement is done in triplicate, and the dots represent different individuals (left panel) or experiments (right panel). The insert with paired data displays the *IL6* transcripts in infected cells with or without IFNβ treatment. One-way ANOVA and Tukey multiple comparison tests were performed (* for *P*-value < 0.05; ** for *P*-value < 0.01; and *ns* for not significant). (**B and C**) HeLa cells were incubated with IFNβ and/or *C. trachomatis* for 24 h prior to adding brefeldin A for 6 h. After the treatment, the intracellular level of IL6 was measured by flow cytometry using anti-human IL6-PC7 antibody. The histograms are from one representative experiment (**B**), and the quantification of four experiments is displayed. *P*‐values of Student’s paired *t*‐test are shown when significant (** for *P*-value < 0.01) (**C**).

Primary ecto-cervical epithelial cells were infected with *C. trachomatis*, in the presence or absence of recombinant human IFNβ, and transcript levels for *IL6* were measured 30 hpi. The transcription of *IL6* triggered by *Chlamydia* infection was enhanced by IFNβ treatment, when IFNβ alone had no effect (Fig. 1A, left panel). A similar enhancement by IFNβ stimulation on IL6 transcripts was observed in infected HeLa cells, a widely used cervical epithelial cell line (Fig. 1A, right panel). To test whether this increase in transcription correlated with higher cytokine levels, we used flow cytometry to detect IL6. This experiment required more cells and was therefore conducted in the cervical cell line HeLa. Cells were infected for 30 h in the presence or absence of IFNβ. In the last 6 h of infection, brefeldin A (BFA) was added to the culture medium to block protein secretion. After fixation and permeabilization, IL6 was stained with antibodies coupled to a fluorochrome, and samples were analyzed by flow cytometry. The addition of IFNβ and *Chlamydia* increased the percentage of cells positive for IL6, compared to infection alone (Fig. 1B and C). Consistent with the data obtained by real-time RT-qPCR, IFNβ alone did not elicit IL6 production in HeLa cells (Fig. 1B and C). Similar results were obtained using IFNα (Fig. S1A), and the response was dose dependent (Fig. S1B). Finally, we observed using a *Chlamydia* strain expressing the green fluorescent protein (GFP) that cells positive for IL6 were mostly infected cells (Fig. S1C). These data suggested a synergistic effect of IFN-I on the *Chlamydia*-induced inflammation in primary epithelial cells as well as in HeLa cells. IFNβ was used in the rest of the study.

### The synergy between IFN-I and *Chlamydia* infection requires TLR3 expression

**Fig 2:**
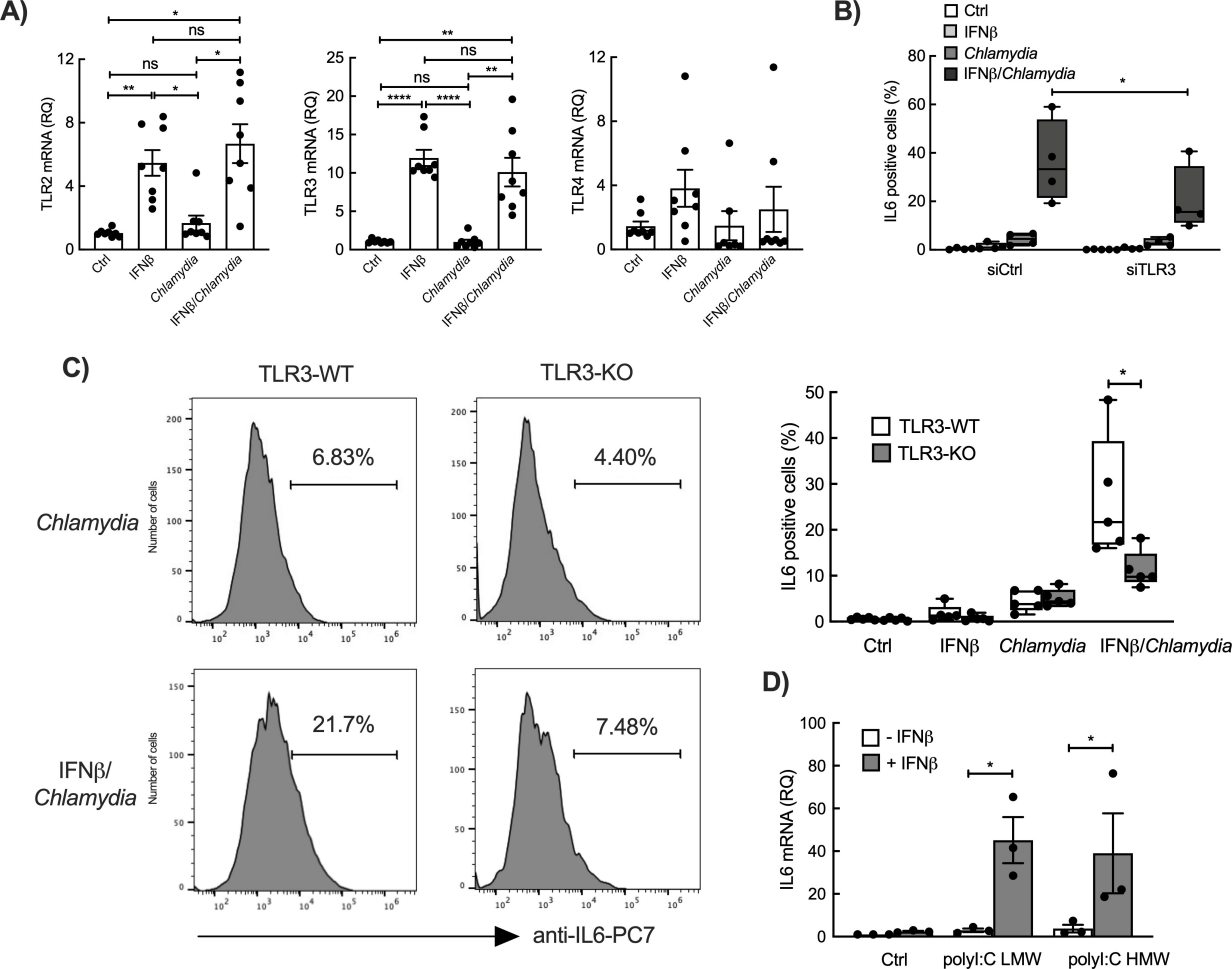
TLR3 mediates the synergy process between IFNβ and *C. trachomatis*. (**A**) Primary cervical epithelial cells were incubated with IFNβ and/or *C. trachomatis*. Twenty-four hours later, the expression of transcripts coding for pattern recognition receptors (PRRs) was measured by real‐time RT-qPCR and normalized to actin transcripts following the 2^−ΔΔCt^ method. The data are presented as relative mRNA levels compared to untreated cells and shown as the mean ± SE with individual values of three independent experiments. Each experiment was conducted in technical triplicate, and each dot represented the data obtained with cells isolated from one individual. One-way ANOVA and Tukey multiple comparison tests were conducted (* for *P* < 0.05, ** for *P* < 0.01, **** for *P* < 0.0001, and *ns* for not significant). (**B**) siRNA was incubated with HeLa cells for 24 h before treatment with IFNβ and/or *C. trachomatis*. Brefeldin A was added 24 hpi for 6 h. Intracellular IL6 protein was measured by flow cytometry using anti-human IL6-PC7 antibody. Data from four independent experiments are shown. The *P*‐value of a Student’s paired *t*‐test is shown (* for *P* < 0.05). (**C**) TLR3-KO cells or parental HeLa cells were infected with *C. trachomatis* in the presence or absence of IFNβ for 30 h before measuring intracellular IL6 levels by flow cytometry. The histograms are from one representative experiment, and data from five independent experiments are displayed (right panel). The *P*‐value of a Student’s paired *t*‐test is shown (* for *P* < 0.05). (**D**) HeLa cells were incubated with IFNβ and/or low/high molecular weight (LMW/HMW) polyI:C (25 μg/mL). Sixteen hours later, the expression of IL6 was measured by real‐time RT-qPCR as described in panel **A**. The data are presented as the mean ± SE with individual values of three independent experiments. Each experiment was conducted in technical triplicate. *P*-values of Student’s paired *t*-tests are shown (* for *P* < 0.05).

Host cells sense invading microorganisms by recognition of pathogen-associated molecular patterns via specific PRRs. We hypothesized that IFN-I might facilitate the detection of the bacteria and thereby enhance the inflammatory response to the infection. To test this hypothesis, we measured the transcription of the genes coding for the PRRs TLR2/3/4, which were previously implicated in the detection of *C. trachomatis* (2–5). IFN-I stimulated the expression of TLR2, TLR3, and TLR4 by primary epithelial cells both in non-infected and infected cells (Fig. 2A). Similar results were observed in HeLa cells (Fig. S2A). Thus, the three PRRs tested could potentially be implicated in the synergistic effect between IFN-I and infection. However, silencing *TLR2* and *TLR4* expression did not affect the level of IL6 production upon *Chlamydia* infection in the presence of IFN-I in HeLa cells (Fig. S2B), while silencing *TLR3* did (Fig. 2B; Fig. S2B), indicating that TLR3 was required. We next generated a *TLR3* KO clone in HeLa cells (Fig. S2C). The production of IL6 in *TLR3* KO cells upon *Chlamydia* infection in the presence of IFNβ was low compared to wild-type (WT) cells (Fig. 2C), confirming that the synergy between IFN-I and infection implicated TLR3. A synergistic effect between IFN-I and polyI:C, a synthetic ligand of TLR3, on IL6 synthesis was also observed (Fig. 2D), confirming that IFN-I can synergize a TLR3 stimulation.

### The synergy between IFN-I and infection requires the PI3K/AKT/mTOR but not the STAT1 signaling pathways

**Fig 3:**
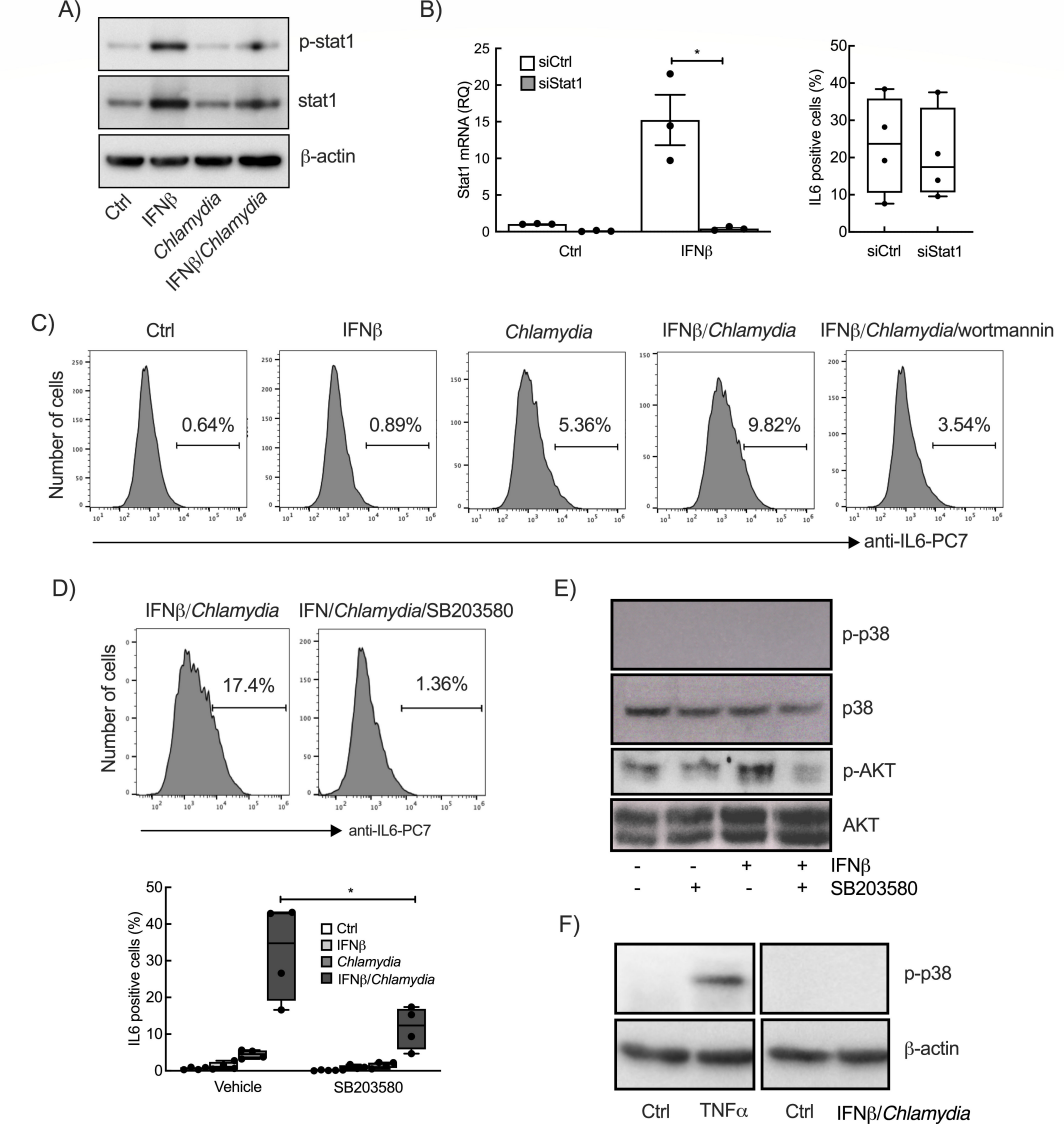
PI3K/AKT but not STAT1 signaling pathways downstream of IFN-I are implicated in the synergy between IFN-I and *C. trachomatis*. (**A**) HeLa cells were incubated with IFNβ and/or *C. trachomatis* for 24 h followed by the detection of activation and expression of STAT1 via immunoblot. The results are representative of three independent experiments. (**B**) siRNA against STAT1 or irrelevant oligonucleotides were transfected into HeLa cells for 24 h prior to incubation with IFNβ alone (left graph) or with IFNβ and *C. trachomatis* (right graph). The transcriptional level of STAT1 (left graph) was measured by real‐time RT-qPCR and normalized to actin transcript following the 2^−ΔΔCt^ method. The data are presented as relative mRNA levels compared to untreated cells and shown as the mean ± SE with individual values of three experiments. The *P*‐value of a Student’s paired *t*‐test is shown (* for *P* < 0.05). Intracellular IL6 protein (right graph) was determined by flow cytometry using anti-human IL6-PC7 antibody. The results of four independent experiments are shown. (**C and D**) HeLa cells were pre-incubated with wortmannin (5 μM) (**C**) or SB203580 (10 μM) (**D**) for 1 h. Cells were then treated with IFNβ and/or *C. trachomatis* for 24 h in the presence of these inhibitors prior to brefeldin A addition for 6 h. Intracellular IL6 expression was analyzed by flow cytometry using anti-human IL6-PC7 antibody. The histograms are representatives of two (**C**) or four experiments (**D**, upper panel), respectively. The results of the four independent experiments are displayed (D, lower panel), with a *P*‐value of a Student’s paired *t*‐test (* for *P* < 0.05). (**E**) The cells were incubated with SB203580 (10 μM) for 1 h before the addition of IFNβ for 30 min. Phosphorylation of p38 and AKT was detected by immunoblot. (**F**) The cells were stimulated with IFNβ and *C. trachomatis* for 24 h or with recombinant human TNFα (10 ng/mL) for 30 min as a positive control, before detection of p38 phosphorylation by immunoblot. The data are representative of three independent experiments.

Which of the IFN-I-activated signaling cascades contributed to the synergy between IFN-I and *C. trachomatis* infection? We first looked at the expression level and phosphorylation of the protein signal transducer and activator of transcription 1 (STAT1). Expression and phosphorylation of STAT1 were increased in cells treated with IFNβ, but not when the cells were infected (Fig. 3A). Moreover, silencing STAT1 had no effect on IL6 production by infected cells treated with IFN-I (Fig. 3B). These data suggest that STAT1 does not contribute to the synergy between IFN-I and *C. trachomatis* on the pro-inflammatory response.

IFN can also signal via the uncanonical PI3K/AKT/p38 pathway (10). Wortmannin, an irreversible inhibitor of PI3Ks, was used to test whether PI3Ks were involved in the synergy between IFN-I and infection. Pre-treatment of cells with wortmannin decreased IL6 production upon infection in the presence of IFN-I (Fig. 3C), indicating that the PI3Ks are part of the implicated signaling cascade(s). SAR405, a specific inhibitor of PI3K/Vps34 (43), had no effect (Fig. S3A), suggesting the involvement of class I and/or class II PI3Ks, but not class III PI3K/Vps34. Protein kinase B (PKB or AKT) and MAP kinase p38 lie downstream of PI3Ks. Incubation of cells with SB203580, a commonly used inhibitor of AKT and p38 (44, 45), prevented IL6 synthesis upon treatment with IFNβ and *Chlamydia* (Fig. 3D). p38 was not activated by IFN-I (Fig. 3E) nor by the combination of IFNβ and *Chlamydia* (Fig. 3F). In contrast, AKT was activated by IFN-I, and its phosphorylation was prevented by SB203580 treatment (Fig. 3E). As positive controls, we verified that recombinant human TNFα induced phosphorylation of p38 (Fig. 3F) and of AKT in a SB203580-sensitive manner (Fig. S3B). These results suggest that in these cells, AKT, but not p38, is involved downstream of IFNβ signaling.

**Fig 4:**
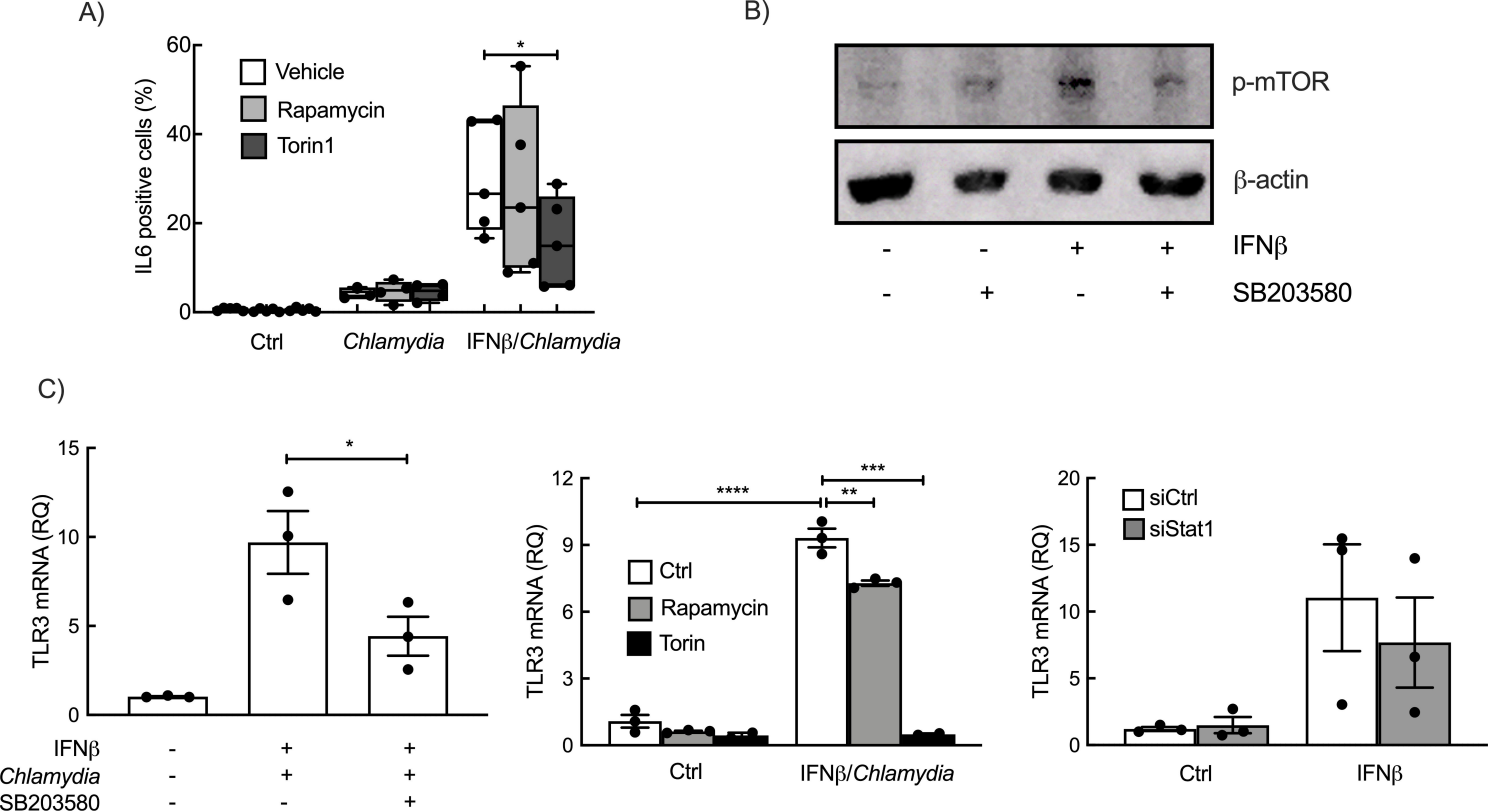
mTOR signaling is involved in the synergy between IFN-I and *C. trachomatis*. (**A**) HeLa cells were pre-incubated with mTOR inhibitors, rapamycin and torin1 (1 μM) for 1 h. Cells were then treated with IFNβ and/or *C. trachomatis* for 24 h in the presence of these inhibitors prior to brefeldin A addition for 6 h. Intracellular IL6 expression was analyzed by flow cytometry using anti-human IL6-PC7 antibody. The results of five independent experiments are displayed with a *P*‐value of a Student’s paired *t*‐test (* for *P* < 0.05). (**B**) HeLa cells were incubated with SB203580 (10 μM) for 1 h before the addition of IFNβ for 30 min. Phosphorylation of mTOR was detected by immunoblot, and β-actin was used as a loading control. The results are representative of three independent experiments. (**C**) HeLa cells were treated with pharmacological inhibitors (left and middle panels) or transfected with siRNA (right panel) as described in Fig. 3, followed by IFNβ and/or *C. trachomatis* treatment for 24 h. TLR3 transcripts were measured by real-time RT-qPCR as above. Data from three independent experiments and *P*‐values of Student’s paired *t*‐test are shown (*P < 0.05, **P < 0.01, ***P < 0.001, and ****P < 0.0001).

We also explored the implication of the kinase mTOR in the signaling cascade(s) linking IFN-I and *C. trachomatis* infection to cytokine secretion. Rapamycin, a specific inhibitor of mTOR complex 1 (mTORC1) (46), had no effect on IL6 synthesis (Fig. 4A). In contrast, torin1, which blocks both mTORC1 and mTORC2, markedly decreased IFNβ/*Chlamydia*-induced inflammation measured on transcript (Fig. S3C) and protein (Fig. 4A) levels. These data indicate that mTORC2 is required for the synergy between IFN-I and *C. trachomatis*. mTOR phosphorylation triggered by IFN-I was attenuated in the presence of SB203508, consistent with its position downstream of PI3K/AKT (10) (Fig. 4B).

Altogether, we conclude that the uncanonical PI3K/AKT/mTORC2, but not canonical IFN signaling STAT1, contributes to the exacerbation of inflammation by IFN-I upon *C. trachomatis* infection in epithelial cells.

### Inhibition of PI3K/AKT and mTOR prevented the upregulation of TLR3 expression

If the synergy between infection and IFN-I is mediated by the upregulation of *TLR3* transcription, inhibition of the signaling pathway downstream of IFN-I is expected to hinder the upregulation of *TLR3* transcription. Indeed, pretreatment of epithelial cells with the AKT inhibitor SB203580 attenuated the upregulation of TLR3 (Fig. 4C). A similar attenuation was detected when using rapamycin, and the effect of Torin1 was even stronger (Fig. 4C). Silencing STAT1 had no effect (Fig. 4C), confirming that the canonical pathway downstream of IFN-I is not involved. These results support the conclusion that the synergy between infection and IFN-I is mediated by the upregulation of TLR3 downstream of PI3K/AKT/mTORC2 signaling.

### The ERK pathway is stimulated downstream of TLR3

We next investigated the signaling intermediates between TLR3 activation and *IL6* expression. TLR3 signaling generally triggers MAPKs, nuclear factor kappa-light-chain-enhancer of activated B cells (NF-κB), and interferon regulatory transcription factor 3 (IRF3) activation to induce the expression of inflammatory cytokines (47, 48). NF-κB is a transcription factor playing a central role in inflammation. p65, one predominant subunit of NF-κB heterodimers, translocates to the nucleus upon activation (49). We tested whether NF-κB was activated by *Chlamydia* infection upon IFN-I treatment using HeLa cells expressing a fusion between p65 and GFP. IL1β was used as a positive control for nuclear translocation of p65-GFP (Fig. S4A, left panels). In contrast, p65-GFP remained cytosolic in *Chlamydia*-infected cells (50), even in the presence of IFN-I (Fig. S4A, middle and right panels). These data indicate that NF-κB is not implicated in the pro-inflammatory response to *Chlamydia* infection, even in synergy with IFN-I. Furthermore, silencing *IRF3* did not abolish the synergy between infection and IFN-I on IL6 expression (Fig. S4B), indicating that the TLR3-IRF3 cascade was also not involved.

**Fig 5:**
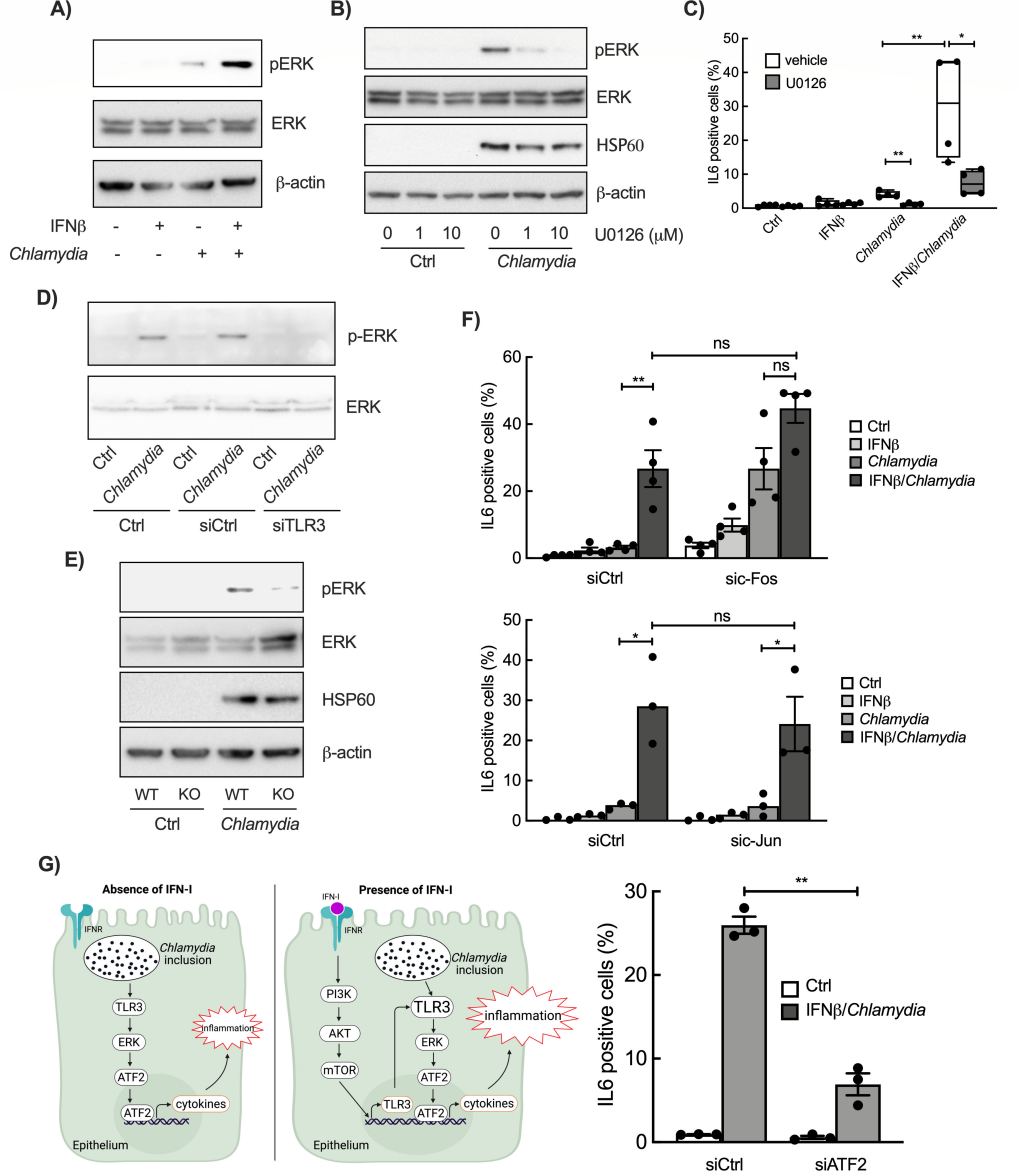
TLR3 modulates the host inflammatory response to the infection through ERK-ATF2. (**A**) HeLa cells were treated with IFNβ and/or *C. trachomatis* for 24 h. The activation of ERK and its expression were determined by immunoblot using anti-phosphorylated ERK and anti-ERK antibodies, respectively. The blots are representatives of three experiments. (**B**) The cells were pre-treated with U0126 at the indicated concentrations. One hour later, HeLa cells were infected with *C. trachomatis* for 24 h, still in the presence of U0126, followed by the detection of ERK phosphorylation as in panel **A**. The blots are representatives of three experiments. (**C**) After 1 h pre-treatment with U0126 (10 μM), HeLa cells were incubated with IFNβ and/or *C. trachomatis* for 24 h in the presence of U0126, prior to adding brefeldin A for 6 h. Intracellular levels of IL6 were measured by flow cytometry using anti-human IL6-PC7 antibody. The results of four independent experiments and the *P*-values of Student’s paired *t*‐tests are shown (*P < 0.05 and **P < 0.01). (**D**) HeLa cells were transfected with control siRNA or siRNA (final concentration 30 nM) against human TLR3 for 24 h before *C. trachomatis* infection for an additional 24 h. The activation and expression of ERK were determined as in panel A. The blots are representative of three experiments. (**E**) HeLa WT cells and TLR3-KO clone were infected with *C. trachomatis* for 24 h before determining ERK activation and expression. The data are representatives of three experiments. (**F**) siRNA against c-Fos (upper panel), c-Jun (middle panel), ATF2 (bottom panel), or irrelevant oligonucleotides were transfected into HeLa cells for 24 h prior to incubation with IFNβ and/or *C. trachomatis* for 24 h. Intracellular levels of IL6 were measured as in panel **C**. The results of three or four independent experiments and the *P*-values of Student’s paired *t*‐tests are shown (*P < 0.05 and **P < 0.01). (**G**) Graphical summary created by BioRender. ns, not significant.

*Chlamydia* infection induced phosphorylation of the MAPK/ERK (Fig. 5A), but not p38 (Fig. 3E). IFN-I alone did not activate ERK, but its presence enhanced *Chlamydia*-induced ERK phosphorylation (Fig. 5A). Application of U0126, an inhibitor of MAP/ERK kinase (MEK), reduced ERK phosphorylation induced by *Chlamydia* infection (Fig. 5B). Pre-incubation of HeLa cells with this inhibitor also alleviated the synergistic effect of IFN-I and infection on IL6 production (Fig. 5C), without blocking *TLR3* transcription (Fig. S4C). In addition, silencing TLR3 also blocked ERK phosphorylation upon infection (Fig. 5D). Consistently, *Chlamydia*-driven ERK activation was reduced in the TLR3-KO cells (Fig. 5E). Altogether, these data converge to place the activation of the MAPK/ERK downstream of TLR3 activation upon *Chlamydia* infection.

Finally, we tested the potential involvement of activator protein 1 (AP-1), downstream of ERK. AP-1 is a transcription factor with a structure of homo- or heterodimer composed of multiple family members, including activating transcription factors (ATFs), c-Jun, and c-Fos (51). We observed that silencing ATF2 significantly reduced IL6 synthesis upon infection in the presence of IFN-I, while silencing c-Jun or c-Fos had no effect (Fig. 5F; Fig. S5). These results suggest that the AP-1 transcription factor family member ATF2 controls *IL6* transcription downstream of TLR3-ERK signaling (Fig. 5G).

### dsRNA is detected in *Chlamydia*-infected cells

**Fig 6:**
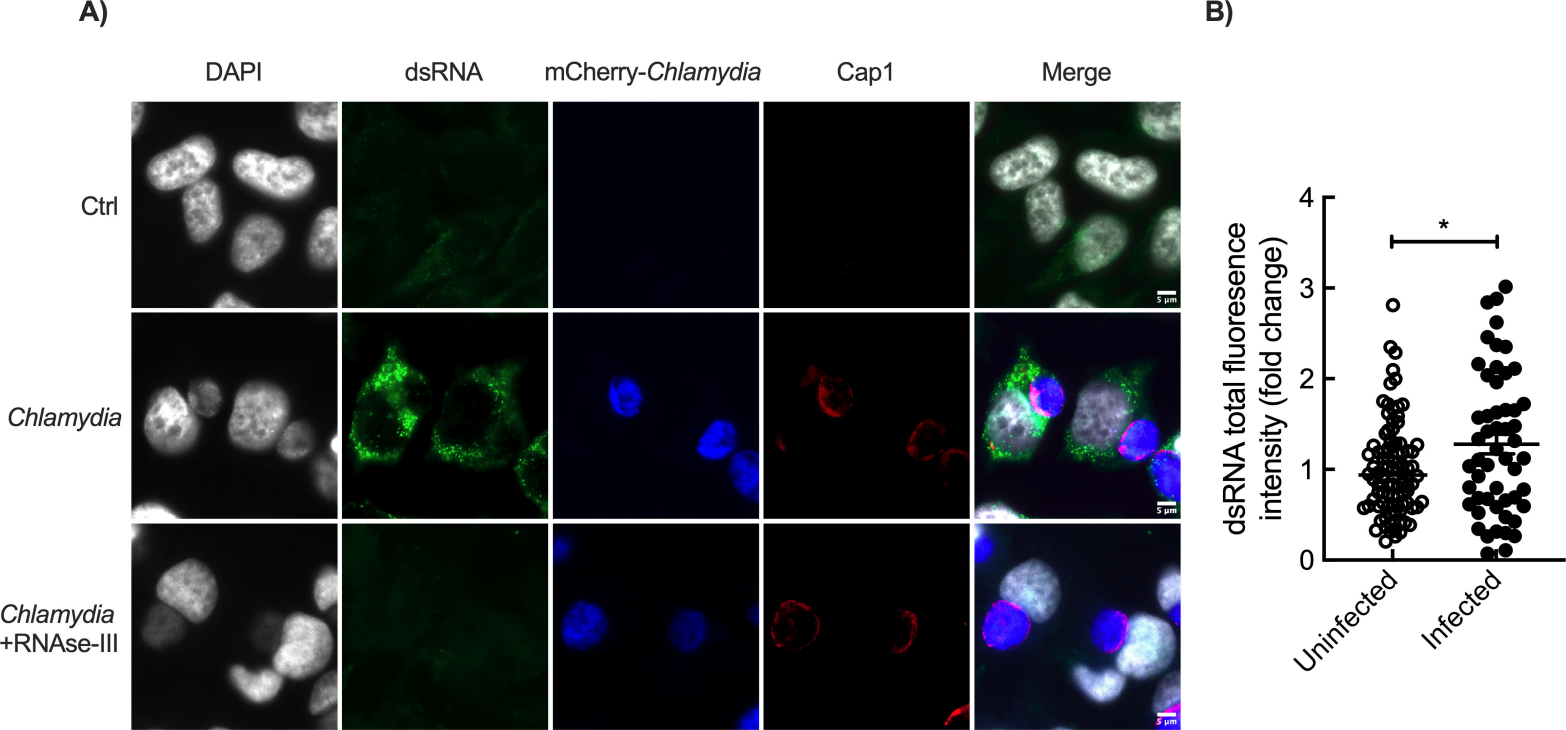
dsRNA accumulates in *Chlamydia*-infected cells. (**A**) HeLa cells were infected with mCherry-expressing bacteria (MOI = 0.3) for 30 h, followed by fixation and immunostaining. DNA was stained with DAPI (gray), the inclusion membrane was labeled with an antibody against the bacterial protein Cap1 (red), and the dsRNA was stained with J2 antibody (green). The mCherry signal is displayed in blue. In the lower panels, the cells were incubated with RNase-III for 30 min before immunostaining. The images are representative of three independent experiments. (**B**) dsRNA fluorescence intensity in the cytoplasm of infected or non-infected cells was quantified as described in the Materials and Methods section, and the *P*-value of a Student’s unpaired *t*‐test is shown (*P < 0.05).

Double-stranded RNA (dsRNA) is the only known trigger of TLR3 activation. To determine whether dsRNA is produced during *C. trachomatis* infection, we stained infected coverslips with the mouse monoclonal J2 antibody against dsRNA and with the rabbit polyclonal antibody against the inclusion protein Cap1. dsRNA was detected in the cell cytoplasm and was more abundant in infected cells (Fig. 6; Fig. S6). To control for the specificity of this staining, cells were incubated before staining with a dsRNA-specific RNase, i.e., RNase-III. Treatment led to a complete disappearance of the dsRNA-associated signal, while the Cap1 signal remained (Fig. 6A). These data indicate that dsRNA is produced during *Chlamydia* infection and could serve as a trigger for TLR3 activation. The observations that the *C. trachomatis* plasmid encodes antisense RNAs which are complementary to open reading frame 8 of the plasmid (52), and that a plasmid-deficient strain induces less pro-inflammatory response than WT bacteria (53), suggest that the plasmid might be a source of dsRNA. To test this possibility, cells were infected with WT or plasmid-less *Chlamydia* (25667R strain), and the dsRNA signal was compared. Similar levels of dsRNA were detected in both conditions, indicating that plasmid-derived dsRNA does not contribute significantly to the dsRNA signal (Fig. S6B and C).

## DISCUSSION

This study examined the effect of IFN-I on the inflammatory response of epithelial cells to *Chlamydia* infection. Treatment of cervical epithelial cells, either primary cells or cancer-derived HeLa cells, with IFN-I strongly increased the synthesis of the pro-inflammatory cytokine IL6 upon *C. trachomatis* infection. This synergistic effect was accounted for by an increase in the expression of the PRR TLR3 upon IFN-I treatment, enhancing *Chlamydia* detection. We determined which signaling cascades, upstream and downstream of TLR3 expression, were implicated.

IFN-I was supplied in the culture medium at nanomolar concentration (2.5 ng/mL or 125 nM), in the range of concentrations detected in the serum of patients with tuberculosis, influenza, or COVID-19 infection, ranging from femtogram per milliliter to nanogram per milliliter (51, 54–56). Almost all cells in the body synthesize IFN-I (11), although immune cells such as plasmacytoid dendritic cells constitute the major source. Upon *Chlamydia* infection, IFN-I secretion has been detected in cultures of HeLa cells, primary human Sertoli cells of the male reproductive system, and oviduct epithelial cells of mice, indicating that autocrine signaling may occur (19, 21, 23, 57). Consistently, using human fallopian tube-derived organoids and human genital serovar of *C. trachomatis,* Kessler et al. reported that *Chlamydia* infection strongly activated the IFN-I cascade pathway (20). Immune cells activated by the infection likely relay and amplify IFN-I synthesis. However, IFN-I is usually not detected in the plasma of patients with *Chlamydia* infection, possibly because of its short half-life (approximately 1–3 h) (58, 59). Also, the peak of IFN-I induction upon *Chlamydia* infection might be missed, and/or IFN-I synthesis might be local and undetectable in the serum or cervical secretions.

We observed that IFN-I stimulated STAT1 expression, and this effect was impaired in cells infected with *Chlamydia*, consistent with other studies reporting an inhibition of STAT1 signaling by *Chlamydia* infection (60, 61), and with the absence of effect of STAT1 silencing on the synergy between IFN-I and *Chlamydia* in our study. SB203580 attenuated the synergy between IFN-I and *Chlamydia* as well as the expression of TLR3. Generally, SB203580 inhibits MAPK p38 kinase (IC_50_ ~ 0.5 µM) and PKB (AKT), another known downstream signal of PI3K (IC_50_ ~ 5 µM) (45). We used SB203580 at 10 μM, which inhibits both kinases. By western blot, we detected phosphorylation of AKT but not of p38 upon *Chlamydia* infection, indicating that, in our cellular model, IFN-I promotes TLR3 expression via AKT stimulation.

Using primary human epithelial cells, we observed that IFN-I increased the expression of *TLR2*, *TLR3*, and *TLR4. TLR3* upregulation by IFN-I was also observed in lung epithelial A549 cells and human umbilical vein endothelial cells (62). IFNγ also enhanced TLR3 expression in keratinocytes (63). Interestingly, *Chlamydia* infection alone did not trigger the transcription of *TLR3* and showed a tendency to dampen the induction of its transcription upon IFN-I stimulation, indicating that the bacteria may partially counteract IFN-I stimulation (Fig. 2A; Fig. S2A). Another study showed that *C. trachomatis* infection elicited TLR3 expression in primary Sertoli cells (57). This increase might rely on IFN-I and an autocrine pathway, as the infection also significantly increased IFNβ production (57). In our model, silencing or knocking out *TLR3*, but not two other TLRs, attenuated the synergistic inflammation to IFN-I and infection, indicating that TLR3 is a key mediator. Supporting this conclusion, knocking out *TLR3* has been shown to severely diminish the immune response to *Chlamydia* infection of mouse and human oviduct epithelial cells, including IL6 secretion (3, 64). Interestingly, TLR3 activation itself triggered IFN-I production in murine oviduct epithelial cells infected with *C. muridarum* (65), suggesting a positive feedback loop between TLR3 and IFN.

What activates TLR3 during *C. trachomatis* infection? TLR3 is mainly known for its ability to recognize dsRNA associated with viral infection. Recognition of poly(I:C), a synthetic mimic of dsRNA, by TLR3 triggers a strong induction of inflammatory cytokines by macrophages via activation of the NF-κB cascade (41). A different signaling cascade is triggered in the context of *Chlamydia* infection, as we and others observed a lack of activation of NF-κB (50, 66). Here, we show an increase in dsRNA levels in the cytoplasm of *Chlamydia*-infected cells. Whether this dsRNA is released by the bacteria or by the host upon *C. trachomatis* infection remains to be determined. Mammalian cells express endogenous TLR3 activators. Short interfering RNA and mRNA are potential ligands of TLR3, likely through the formation of secondary structures containing stretches of dsRNA (67–69). While several mechanisms ensure that these “self” dsRNAs do not elicit an immune response, failure to do so was reported in different pathological contexts (70–72). Whether *Chlamydia* infection results in endogenous dsRNA leakage into the cytoplasm remains to be elucidated. Alternatively, an increase in cytoplasmic dsRNA content might be of bacterial origin. Similar levels of dsRNA were detected in cells infected with a plasmid-less strain as with a WT strain, indicating that plasmid-derived RNAs are not implicated.

Transcription factor AP-1 complexes are key mediators of innate immune responses. The typical heterodimer form of this complex, c-Jun/c-Fos, is not involved in TLR3-ERK-mediated inflammatory response to *Chlamydia* infection. In contrast, silencing the other AP-1 component ATF2 significantly decreased the synergistic effect between IFN-I and infection. Since silencing c-Jun did not phenocopy ATF2 silencing, we hypothesize that ATF2 homodimers, rather than c-Jun/ATF2 heterodimers (73), might be implicated.

In this study, IFN-I was shown to exacerbate *Chlamydia*-induced inflammation in epithelial cells. By exacerbating the pro-inflammatory response of epithelial cells, IFN-I might contribute to the hyperinflammation experienced by some individuals. Indeed, other studies converge to a deleterious effect of IFN-I production during *C. trachomatis* infection (22, 26–28). The signaling pathways we uncovered can serve as a starting point toward novel therapeutic strategies to alleviate tissue damage after *Chlamydia* infection.

## SUPPLEMENTAL MATERIAL

Fig. S1 mbio.00527-26-s0001.tiff IFNα exacerbates *Chlamydia*-induced inflammation.

Fig. S2 mbio.00527-26-s0002.tiff Expression of PRRs and their roles in the synergy between IFNβ and *Chlamydia*.

Fig. S3 mbio.00527-26-s0003.tiff PI3K/Vps34, MAPK/p38, and mTOR complex 1 are not implicated in the synergy between IFN-I and *Chlamydia*.

Fig. S4 mbio.00527-26-s0004.tiff NF-κB and IRF3 are not implicated in the synergy between IFNβ and *C. trachomatis* infection.

Fig. S5 mbio.00527-26-s0005.tiff Efficacy of the siRNA at silencing c-Fos, c-Jun, and ATF2 in HeLa cells.

Fig. S6 mbio.00527-26-s0006.tiff dsRNA accumulation in *Chlamydia*-infected cells.

Legends mbio.00527-26-s0007.docx Legends for the supplemental figures

Table S1 mbio.00527-26-s0008.docx Primer sequences.

ASM does not own the copyrights to Supplemental Material that may be linked to, or accessed through, an article. The authors have granted ASM a non-exclusive, world-wide license to publish the Supplemental Material files. Please contact the corresponding author directly for reuse.
