# Supplementary figures and images for: IFN-I exacerbates the inflammatory response of epithelial cells to Chlamydia trachomatis infection by enhancing TLR3 expression
Source: mBio. 2026 Jun 15;17(7):e00527-26. doi: 10.1128/mbio.00527-26 (PMC13344013; doi:10.1128/mbio.00527-26)

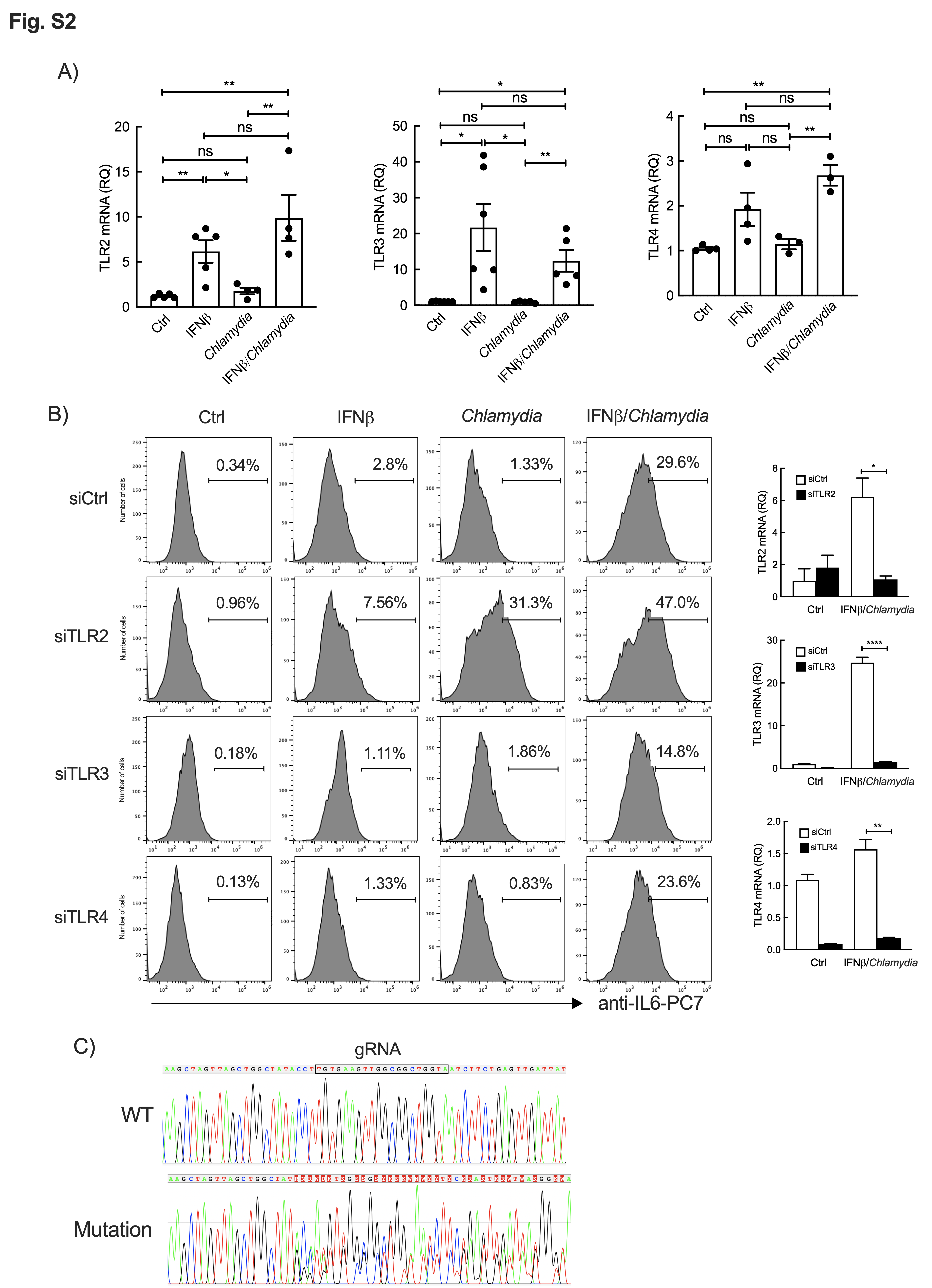

Supplement: Fig. S2 — Expression of PRRs and their roles in the synergy between IFNβ and Chlamydia. [file mbio.00527-26-s0002.tiff]

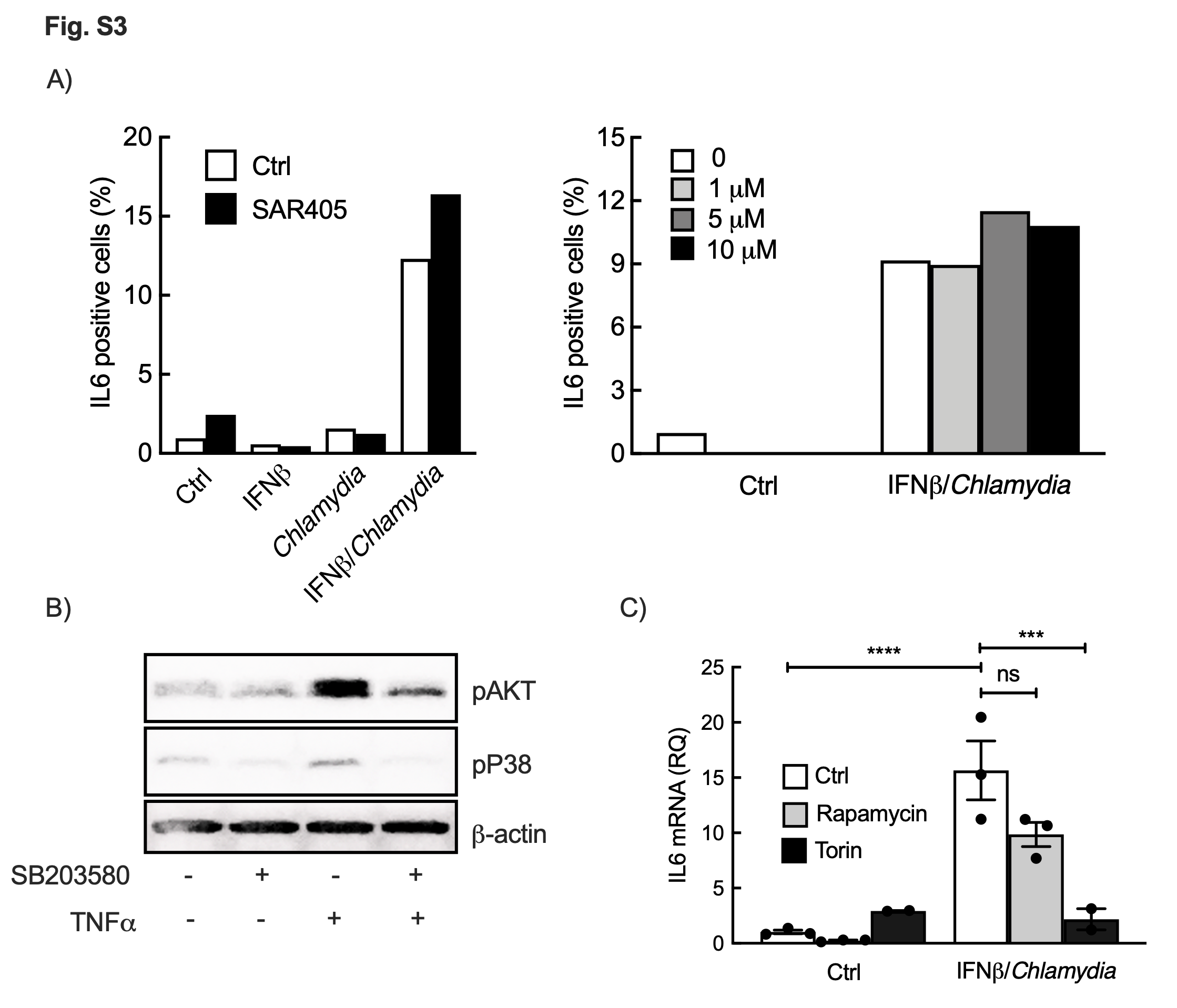

Supplement: Fig. S3 — PI3K/Vps34, MAPK/p38, and mTOR complex 1 are not implicated in the synergy between IFN-I and Chlamydia. [file mbio.00527-26-s0003.tiff]

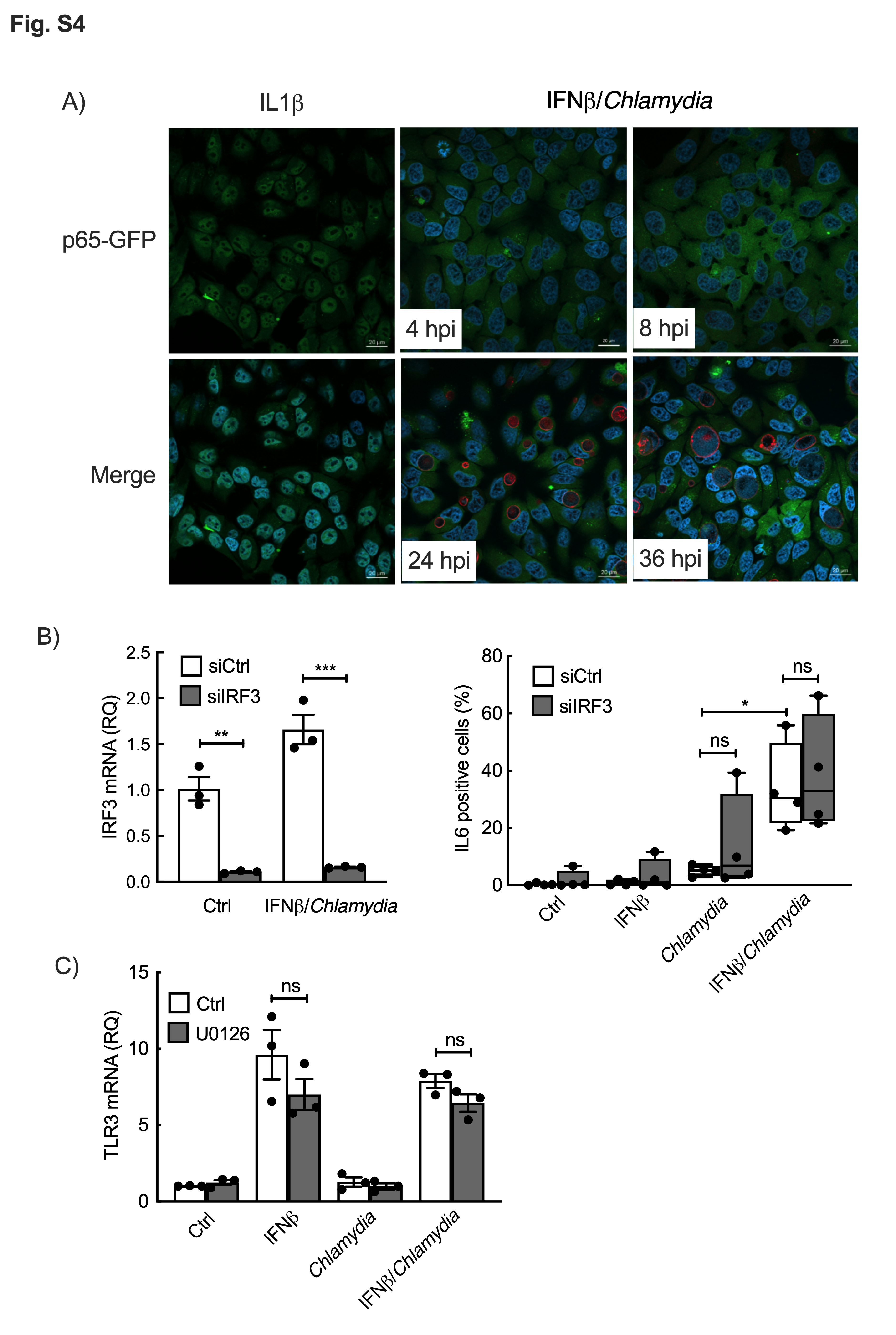

Supplement: Fig. S4 — NF-κB and IRF3 are not implicated in the synergy between IFNβ and C. trachomatis infection. [file mbio.00527-26-s0004.tiff]

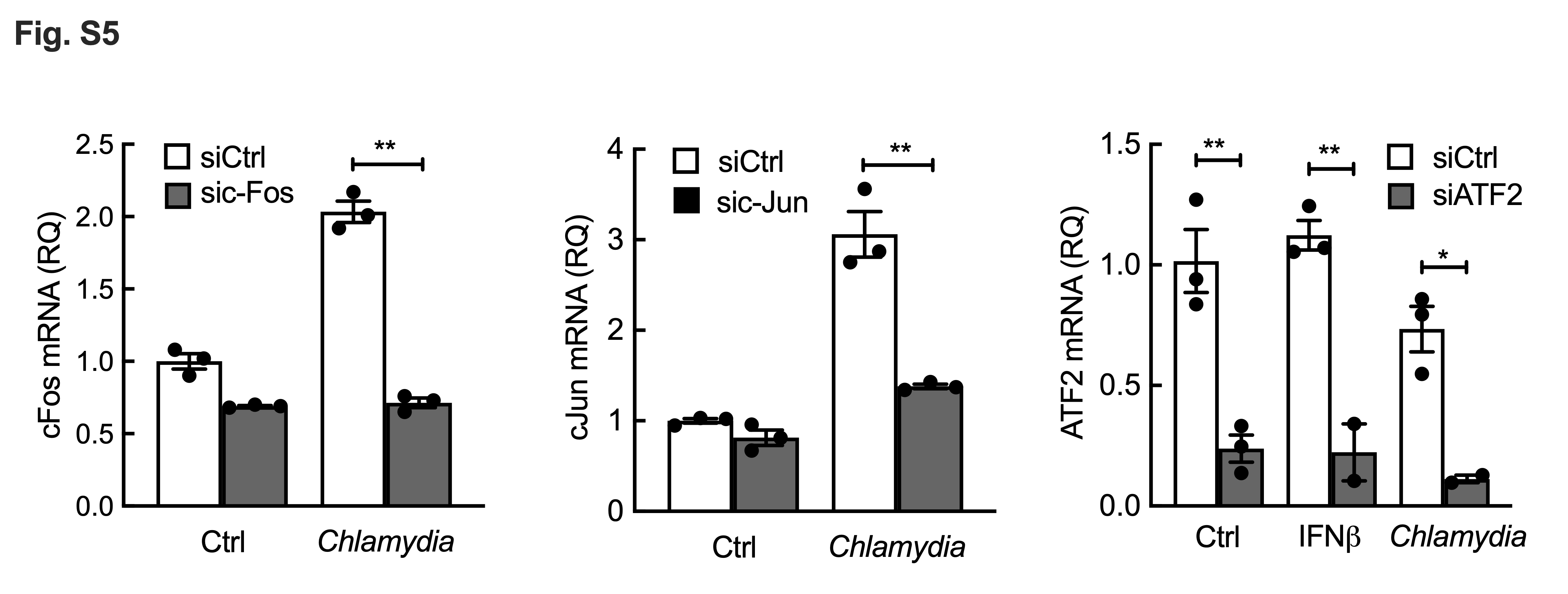

Supplement: Fig. S5 — Efficacy of the siRNA at silencing c-Fos, c-Jun, and ATF2 in HeLa cells. [file mbio.00527-26-s0005.tiff]

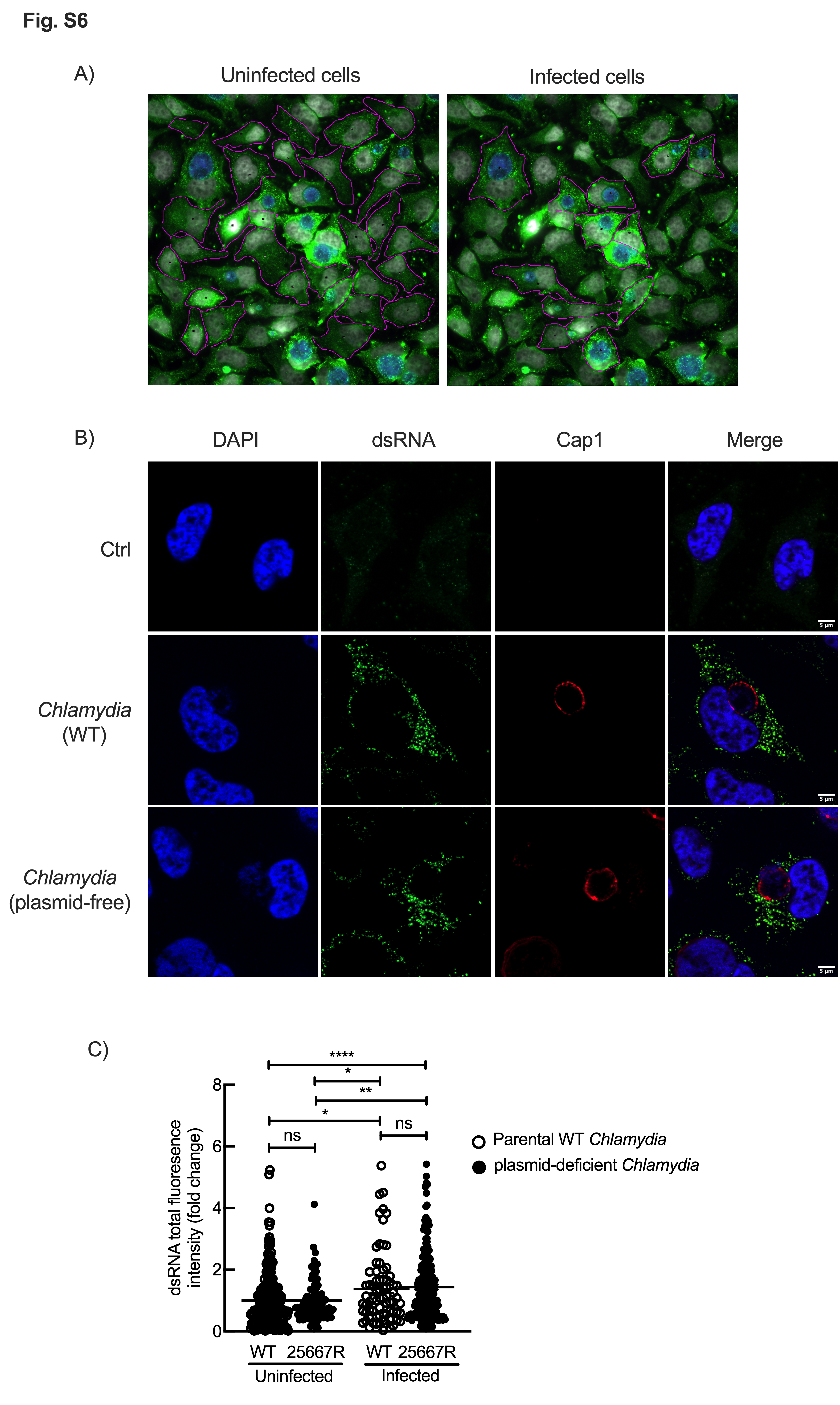

Supplement: Fig. S6 — dsRNA accumulation in Chlamydia-infected cells. [file mbio.00527-26-s0006.tiff]
