# Supplementary material for: IFN-I exacerbates the inflammatory response of epithelial cells to Chlamydia trachomatis infection by enhancing TLR3 expression
Source: mBio. 2026 Jun 15;17(7):e00527-26. doi: 10.1128/mbio.00527-26 (PMC13344013; doi:10.1128/mbio.00527-26)
Supplement: Legends — for the supplemental figures [file mbio.00527-26-s0007.docx]

**LEGENDS OF SUPPLEMENTARY FIGURES**

Fig. S1 IFNα exacerbates *Chlamydia*-induced inflammation. (A) HeLa cells were incubated with IFNα and/or *C. trachomatis* for 24 h before adding brefeldin A for 6 h. Intracellular IL6 protein was determined by flow cytometry using anti-human IL6-PC7 antibody. The results of four independent experiments and the p-value of a Student’s paired t-test are shown (* p<0.05). (B) The same as in A, using IFNα at the indicated concentration. Data are representative of two experiments. (C) HeLa cells were incubated with IFNβ and/or *C. trachomatis* for 24 h prior to adding brefeldin A for 6 h. Intracellular IL6 protein was determined by flow cytometry using anti-human IL6-PC7 antibody. The plots are from one experiment representative of three.

Fig. S2 Expression of pattern recognition receptors (PRRs) and their roles in the synergy between IFNβ and *Chlamydia*. (A) HeLa cells were treated with IFNβ and/or *C. trachomatis* for 24 h, followed with RNA extraction. The transcriptional level of *TLR2*, *TLR3* and *TLR4* was determined by rea-time RT-qPCR and normalized to actin transcripts following the 2^-ΔΔCt^ method. The data are presented as relative mRNA levels compared to untreated cells. Each dot represents the result from one experiment. Student’s paired t-tests were applied for the statistical analysis (* for p< 0.05, ** for p<0.01, ns for not significant). (B) HeLa cells were incubated with siRNA for 24 h prior to treatment with IFNβ and/or *C. trachomatis* for another 24 h. Brefeldin A was added for 6 h before examining the intracellular IL6 by flow cytometry using anti-human IL6-PC7 antibody. The mRNA levels of the indicated PRRs were determined by quantitative RT-PCR (right panel). The histograms are the representatives of two individual experiments. The RT-PCR experiments are representatives of two independent experiments (right panel). P‐values of Student's paired t‐test are shown (* for p< 0.05, ** for p<0.01 and **** for p<0.0001). (C) Sequencing data for TLR3-WT and for the knock-out clone generated by CRISPR-Cas9.

Fig. S3 PI3K/Vps34, MAPK/p38 and mTOR complex 1 are not implicated in the synergy between IFN-I and *Chlamydia*. (A) HeLa cells were pre-treated with SAR405 at 400 nM (left panel) or at the indicated concentration (right panel) for 1 h before treating with IFNβ and/or *C. trachomatis* for 24 h in the presence of inhibitor. Brefeldin A was then added for 6 h prior to fixation and measure of IL6 levels by flow cytometry. The data are representative of two individual experiments. (B) HeLa cells were pre-treated or not with SB203580 (10 μM) for 1 h before incubating with TNFα (10 ng/ml) for 30 min, followed by p38 and AKT phosphorylation detection by immunoblot. The results represent two independent experiments. (C) HeLa cells were pre-incubated with the inhibitor rapamycin (1 μM) or torin1 (1 μM) for 1 h before addition of IFNβ and *C. trachomatis* for 40 h. After treatment, IL6 transcripts were measured by real‐time RT-qPCR and normalized to actin transcript following the 2^-ΔΔCt^ method. The data of three independent experiments and p‐values of Student's paired t‐test are shown (*** for p< 0.001, **** for p<0.0001, ns for not significant).

Fig. S4 NF-κB and IRF3 are not implicated in the synergy between IFNβ and *C. trachomatis* infection. (A) p65-GFP expressing HeLa cells were seeded on coverslip and incubated with recombinant human IL1β (10 ng/ml) for 30 min (left panel), or with IFNβ and *C. trachomatis* for the indicated time (middle & right panels). After cell fixation and permeabilization DNA was stained with DAPI (blue) and the inclusion membrane was labeled with an antibody against the bacterial protein Cap1 followed with Alexa647-conjugated secondary antibody (red). The p65-GFP signal is displayed in green. The images are representatives of three experiments. (B) HeLa cells were incubated with siIRF3 or irrelevant oligonucleotide for 24 h. The cells were then treated with IFNβ and *C. trachomatis* for 30 h before analysis of IRF3 transcription by RT-PCR (left panel) and intracellular IL6 by flow cytometry (right panel). The result from three (left) and four (right) independent experiments and the p-values of Student’s paired t-test are shown (* for p<0.05, ** for p<0.01, *** for p<0.001, and ns for not significant). (C) HeLa cells were pre-incubated with the inhibitor U0126 (10 μM) for 1 h before adding IFNβ and/or *C. trachomatis* for 40 h. TLR3 transcripts were measured by quantitative RT-PCR as described above. The data are presented as mean ± SE of three independent experiments. The p-values of Student’s paired t-tests are shown (ns: not significant).

Fig. S5 Efficacy of the siRNA at silencing c-Fos, c-Jun and ATF2 in HeLa cells. HeLa cells were transfected with control siRNA or siRNA against human c-Fos, c-Jun or ATF2 (final concentration 30 nM) for 24 h before *C. trachomatis* infection or IFNβ treatment for 24 h. Transcription levels for the indicated genes were measured by real‐time RT-qPCR and normalized to actin transcript following the 2^-ΔΔCt^ method. The data of three independent experiments and p‐values of Student's paired t‐tests are shown (* p< 0.05, ** p<0.01).

Fig. S6 dsRNA accumulation in *Chlamydia*-infected cells. (A) Example of automated detection of “Non-infected” and “Infected” cells. Cells were segmented (purple line) and the presence of an inclusion within the cell identified infected cells. Quantification of the dsRNA signal was performed on the cytoplasm (excluding the signal overlapping with nuclei or inclusions), see Materials and Methods for details. (B) HeLa cells were infected with plasmid-deficient or WT bacteria (MOI=0.3) for 30 h, followed by fixation and immunostaining. DNA was stained with DAPI (blue), the inclusion membrane was labeled with an antibody against the bacterial protein Cap1 (red) and the dsRNA was stained with J2 antibody (green). The images are representatives of three independent experiments. C) dsRNA fluorescence intensity in the cytoplasm of infected or non-infected cells was quantified as described in the Methods section. One-way ANOVA and Tukey multiple comparison tests were conducted (* for p < 0.05, ** for p <0.01, **** for p <0.0001, and *ns* for not significant).
