# Supplementary material for: IFN-I exacerbates the inflammatory response of epithelial cells to Chlamydia trachomatis infection by enhancing TLR3 expression
Source: mBio. 2026 Jun 15;17(7):e00527-26. doi: 10.1128/mbio.00527-26 (PMC13344013; doi:10.1128/mbio.00527-26)
Supplement: Table S1 — Primer sequences. [file mbio.00527-26-s0008.docx]

Table S1. Sequences of the primers used

|  |  | sense | anti-sense |
| --- | --- | --- | --- |
| 1 | β-actin | GGACTTCGAGCAAGAGATGG | GCAGTGATCTCCTTCTGCATC |
| 2 | IL6 | CACACAGACAGCCACTCACC | CATCCATCTTTTTCAGCCATC |
| 3 | TLR2 | ATCCTCCAATCAGGCTTCTCT | GGACAGGTCAAGGCTTTTTACA |
| 4 | TLR4 | TTTGGACAGTTTCCCACATTGA | AAGCATTCCCACCTTTGTTGG |
| 5 | TLR3* | GTGATGCTTTCTCTTGGTTG | GTTGGCTATGTTGTTGTTGC |
| 6 | TLR3** | GCCTTGTATCTACTTTTGGG | GACTTTTGTCACGACTTCAC |
| 7 | STAT1 | ACAAGGTGGCAGGATGTCTC | AAACGGATGGTGGCAAATG |
| 8 | c-Fos | CCGGGGATAGCCTCTCTTACT | CCAGGTCCGTGCAGAAGTC |
| 9 | c-Jun | TCCAAGTGCCGAAAAAGGAAG | CGAGTTCTGAGCTTTCAAGGT |
| 10 | IRF3 | AAGGAAGGAGGCGTGTTTG | GTGGCTGTTGGAAATGTGC |
| 11 | ATF2 | AATTGAGGAGCCTTCTGTTGTAG | CATCACTGGTAGTAGACTCTGGG |

The primers were used for RT-qPCR* and for screening KO clones**, respectively.
